# The Effectiveness of Student-Led Musculoskeletal and Vascular Ultrasound Workshops at a Single Institution: A Retrospective Survey Analysis

**DOI:** 10.7759/cureus.41902

**Published:** 2023-07-14

**Authors:** Jane J Kim, Jeffrey J Li, Quang Nguyen, Eric Neilson

**Affiliations:** 1 Education, California University of Science and Medicine, Colton, USA; 2 Family Medicine, California University of Science and Medicine, Colton, USA

**Keywords:** peer teaching, resource limited, student tutor, medical school education, point-of-care-ultrasound

## Abstract

Introduction

Point-of-care ultrasound (POCUS) is a rapidly evolving field of diagnostic medicine as its low cost, portability, and versatility have made handheld ultrasound (US) probes an invaluable tool for many modern physicians. Despite US’s benefits as a bedside evaluative tool, many medical schools have not integrated POCUS into their pre-clerkship medical education due to a lack of equipment and faculty. The first objective of our study was to determine whether student tutors (STs) would be effective resources to teach musculoskeletal (MSK) and vascular US to preclinical medical students. The second objective of our study was to determine whether students who previously attended ST-run MSK US workshops perform better in vascular US than those who did not.

Methods

Six POCUS workshops were led by STs after approval from experienced US faculty. These included US workshops on gastrointestinal structures, forearm structures, joint structures, basic echocardiography, and US-guided IV access. We collected data from two of our six workshops. We developed surveys to gauge the confidence and ability of students to perform US after an MSK workshop and US-guided IV access workshop led by STs. We also measured students' US abilities and collected student feedback after our US-guided IV access workshop. We evaluated students’ US competency in US-guided IV access via their ability to correctly position the US probe, angle the needle of insertion, move the probe with the needle, and access the vein based on the accuracy of the movements. We divided student results into two groups: students who previously attended the MSK workshop before attending the US-guided IV access workshop and students who did not attend the MSK workshop before attending the US-guided IV access workshop. We used averages, frequencies, and two-tailed t-tests to analyze the survey responses and US-guided IV access skill assessments.

Results

Fifty percent of first- and second-year surveyed students “agreed,” and 32.4% “strongly agreed” that they felt confident using US after an ST-run MSK workshop. About 29.4% of surveyed students “agreed” and 41.2% “strongly agreed” that they felt comfortable explaining basic US concepts, such as proper probe positioning and echogenicity. The group of students who attended the MSK workshop prior to the peripheral IV workshop scored similarly to the students who did not attend the MSK workshop (14.33±1.03 versus 14.20±0.84 points).

Both groups of students had an average of over 94% accuracy in technique, positioning, angling, moving the US probe, and achieving US-guided venous access after being taught by STs.

Qualitative surveying noted positive student feedback, such as "Teacher was great at guiding us through the procedure." Survey responses also included suggestions on adding and diversifying equipment, such as “[It would be useful to have a] different type of needle to see the difference on ultrasound.”

Conclusion

Based on the high percentages of accuracy and confidence, we found that STs were effective resources to teach MSK and vascular POCUS and that students who attended previous MSK ST-run US workshops had stronger vascular US ability over time compared to those who did not. Our data support the use of STs as US educational resources, especially in institutions without an existing pre-clerkship US curriculum and limited US resources.

## Introduction

Different parts of the data from this article were previously presented as poster presentations at the Experimental Biology Conference 2022 on April 3, 2022, the 53rd Annual Congress of the Korean Society of Ultrasound in Medicine on May 12, 2022, and the American Academy of Family Physicians 2022 National Conference on July 27, 2022.

Point-of-care ultrasound (POCUS) is quickly becoming part of the standard bedside examination in primary care due to its portability, versatility, and low cost [1]. During the COVID-19 pandemic, bedside ultrasound (US) was used to help inform the initiation, escalation, titration, and weaning of respiratory support in patients with respiratory distress [2]. Although US has traditionally been implemented in emergency medicine, obstetrics and gynecology, surgery, and internal medicine, POCUS is now used regularly in many other specialties, such as family medicine [3,4].

Although 75% of medical schools have a structured POCUS curriculum, our medical school, the California University of Science and Medicine (CUSM), did not and had limited resources to do so [5]. The lack of US faculty as well as the need for individualized hands-on practice time have been barriers to medical school US education [6]. However, studies have shown the efficacy of using peer tutors to combat the lack of faculty resources in teaching US at the pre-clerkship level [7]. Some medical schools have initiated student-led education where experienced students form a curriculum to incorporate POCUS into medical education [8,9].

We aimed to create a student-led US workshop curriculum to provide POCUS education not currently available at our medical school. Throughout the year, we conducted workshops focusing on various organ systems such as renal, cardiac, musculoskeletal (MSK), vascular, and gastrointestinal. Our study focuses on the data collected from MSK and vascular workshops. The objective of our study was to determine whether student tutors (STs) would be effective resources to teach MSK and vascular US to preclinical medical students and whether students who previously attended MSK ST-run US workshops perform better vascular US than those who did not by analyzing the outcomes in confidence and accuracy across various workshops.

## Materials and methods

CUSM’s Institutional Review Board granted approval (HS-2021-15). We designed a series of student-led POCUS workshops to teach US techniques, practice US procedures, and increase interest in POCUS for preclinical medical students in the absence of a pre-existing US curriculum. STs underwent two-hour weekly training sessions for two months on US foundations and basic structures, as well as two-hour workshop-specific training prior to all workshops from faculty who were experienced, US physicians. All training sessions were conducted in hands-on three to five student groups with the US faculty. After receiving approval from the US faculty, US STs ran six US workshops open to all pre-clerkship medical students during the 2021-2022 school year. These included US workshops on abdominal structures, forearm structures, joint structures, basic echocardiography, and US-guided venous access. We collected data from two of our six workshops. We surveyed student confidence after the MSK workshop and US-guided IV access workshop. We also measured students' US abilities and collected student feedback after our US-guided IV access workshop. We divided student results into two groups: students who previously attended the MSK workshop before attending the US-guided IV access workshop and students who did not attend the MSK workshop before attending the US-guided IV access workshop.

These workshops were promoted through email to first- and second-year medical students after approval from the US faculty. Post-workshop surveys were prepared for MSK and US-guided IV workshops.

During the MSK workshop, 34 students were surveyed afterward on their confidence using the workshop material and skills with the statements “I feel confident in US after my US training session” and “I feel comfortable explaining basic US concepts,” graded on a five-point Likert scale ranging from “strongly disagree” to “strongly agree.”

During the US-guided intravenous access workshop, 12 students were surveyed on their confidence in their US skills after the workshop. Responses ranged from “disagree” to “agree” for the statements “I know how to position the probe for ultrasound-guided IV access,” “I know the angle to insert the needle for ultrasound-guided IV access,” “I can properly access the vein using ultrasound,” and “I am confident in my overall ability to perform ultrasound-guided IV access.” Students were also tested on their abilities to correctly position the US probe, angle the needle of insertion, move the probe with the needle, access the vein, and their overall technique based on the accuracy of their movements. The students were graded on their performance by STs on-the-spot. Student performance results were stratified based on whether or not they had previous MSK US experience from the previous ST-run workshop. The previous MSK US workshop focused on training students to use US to find and identify different superficial volar arm structures, including the radial nerve, radial artery, median nerve, ulnar artery, and ulnar nerve. The Ultrasound Guided IV Trainer Vascular Phantom models were used during this workshop. Free response feedback was also collected. All survey questions and US workshops were designed by STs and approved by the US faculty.

Statistical tests used were frequencies to determine percentages of responses, averages to determine scores during the US-guided intravenous access workshop, and two-tailed t-tests to determine any significance between how the two groups in the US-guided intravenous access workshop performed.

## Results

Over the eight months since the beginning of the 2021-2022 school year, we held six POCUS workshops for preclinical medical students ranging from 10 to 50 student sign-ups per workshop.

**Figure 1:**
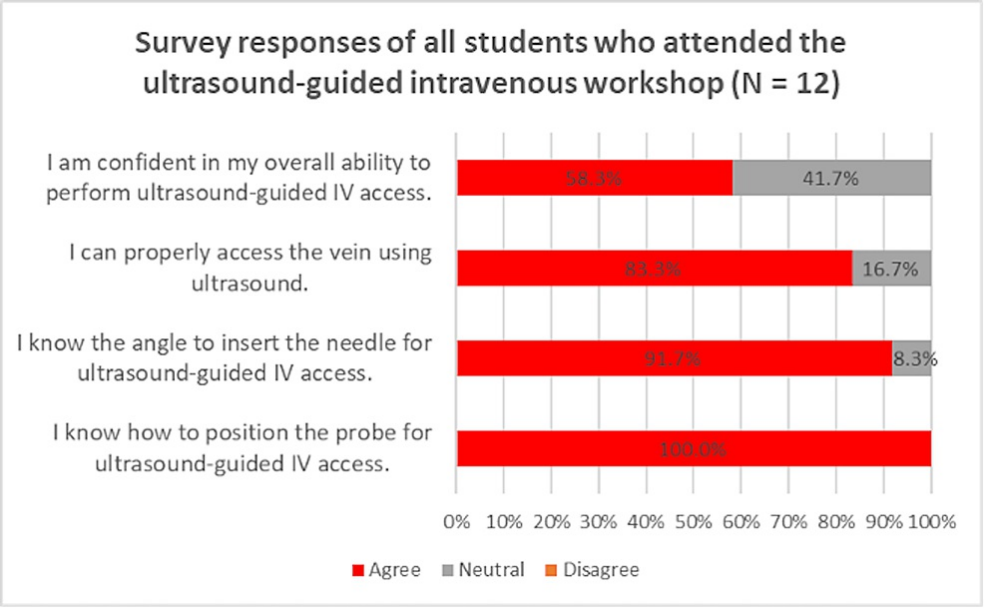
Confidence levels in students who attended the US-guided IV workshop

From the US-guided intravenous access workshop by STs, 58.3% of students agreed that they felt confident in performing US, 83.3% agreed that they can properly access the vein using US, 91.7% agreed that they know the angle to insert the needle for US-guided IV access, and 100% agreed that they know how to position the probe for US-guided IV access (Figure 1). Students were also graded on their US performance after the workshop. Grading criteria are listed in Table 1 and the subsequent results are listed in Table 2. Students were graded out of a total of 15 possible points, with 3 total points possible in each US ability tested. Students who attended our prior ST-led MSK workshop performed slightly better during our US-guided intravenous access workshop on the basis of probe positioning, angle of needle insertion, probe movement with the needle, venous access, and overall technique, although not to a statistically significant extent (Table 2). According to Table 2, the group of students that attended only the peripheral IV workshop had an average score of 14.20±0.84 points out of 15 points based on the scoring criteria from Table 1. The group that attended the MSK workshop prior to the peripheral IV workshop had an average score of 14.33±1.03 out of 15 points.

**Table 1:** Grading criteria for the US-guided IV access workshop

Procedure	0 points	1-2 points	3 points
Position of probe	Student does not position probe transversely/ longitudinally AND does not center needle/vessel.	Student does not position probe transversely/ longitudinally OR does not center needle/vessel.	Student positions the probe transversely/ longitudinally, centering the needle and vessel on the center of the screen.
Angle of needle insertion	Student angles needle <45 degrees during insertion AND >45 degrees after insertion.	Student angles needle ~45 degrees during insertion OR <45 degrees after insertion.	Student has an angle of ~45 degrees during insertion and ~45 degrees or less after penetrating the surface of the IV model.
Probe movement with needle	Student needs to restart the procedure, take out the needle, and reposition the model.	Student loses the needle tip and needs to rescan the model to find the needle tip again.	Student moves the probe after the tip of the needle, moving the probe forward after the white tip of the needle becomes apparent on the screen.
Venous access	Student does not access the vein.	Student accesses the vein but punctures the other side.	Student accesses the vein and does NOT puncture through to the other side.
Overall technique	1) Student does not understand the probe marker in relation to the image. 2) Student is not able to navigate transverse and longitudinal positions. 3) Student is not able to use the image to find the needle.	1) Student understands the probe marker in relation to the image OR 2) Student is able to navigate transverse and longitudinal positions OR 3) Student is able to use the image to find the needle.	1) Student understands the probe marker in relation to the image. 2) Student is able to navigate transverse and longitudinal positions. 3) Student is able to use the image to find the needle.

**Table 2:** Comparison of average scores (max 15 points) of students who attended the MSK workshop prior to the peripheral IV access workshop versus students who did not attend the optional MSK workshop beforehand; post-workshop student results (p = 0.822) MSK: musculoskeletal, IV: intravenous

	Position of probe	Angle of needle insertion	Probe movement with needle	Venous access	Overall technique	Score
Attended only the peripheral IV access workshop (n = 5)	3.00	2.80	2.40	3.00	3.00	14.20±0.84
Attended MSK workshop prior to the peripheral IV access workshop (n = 7)	2.83	3.00	2.67	2.83	3.00	14.33±1.03

**Figure 2:**
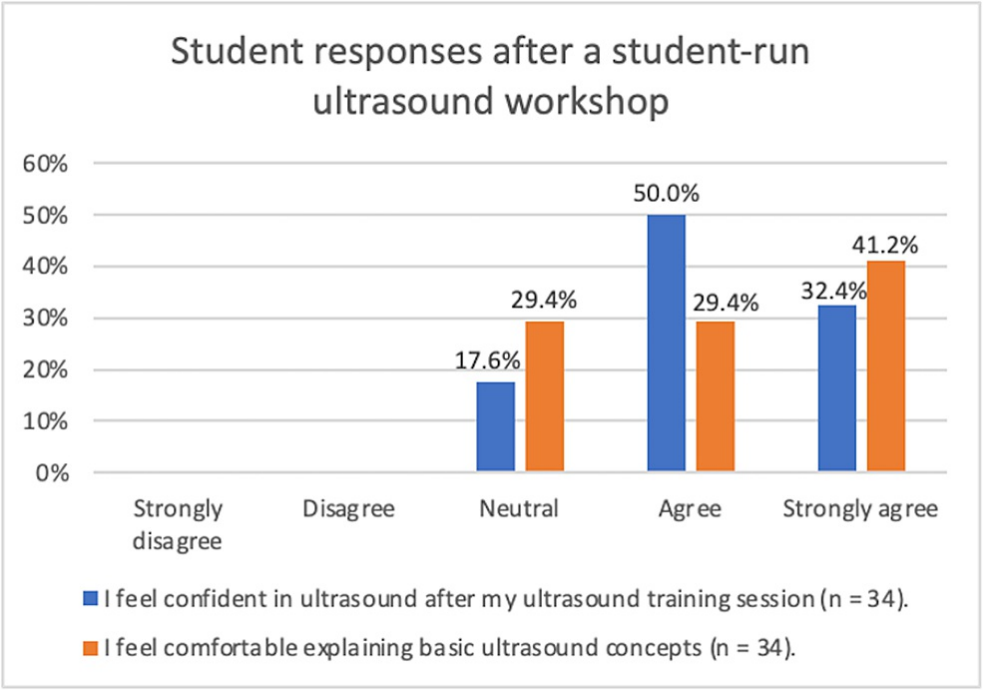
Student responses after an ST-run MSK US workshop

According to Figure 2, 50% of combined first- and second-year surveyed students agreed and 32.4% strongly agreed that they felt confident using US after an MSK workshop. About 29.4% of surveyed students agreed and 41.2% strongly agreed that they felt comfortable explaining basic US concepts, such as proper probe positioning and echogenicity.

Qualitative feedback was collected during the US-guided IV access workshop that demonstrated high student satisfaction in various components of the course as well as a desire to have additional opportunities for US training, with one student commenting they “would love to see this incorporated into the curriculum” (Table 3). Two responses mentioned that incorporating different-sized needles would be helpful in seeing variations.

**Table 3:** Average sample of qualitative student feedback after a US-guided IV access workshop

Average sample of student feedback	"Instructions were clear and ample time was given for practice."	"Instructor was awesome, the video was great, and everyone was super supportive."
	"Teacher was great at guiding us through the procedure."	"The demonstration beforehand helped."
	"Having demonstrations plus practice with guidance made me more confident."	"Great and efficient session. (...) Would love to see this incorporated into the curriculum."
	“[It would be useful to have a] different type of needle to see the difference on ultrasound.”	“Different needle types/sizes [would help] (we only had two).”

## Discussion

Both groups of students who attended a prior US MSK workshop and those who did not perform US-guided IV access had an average of over 94% accuracy in technique, positioning, angling, moving the US probe, and achieving US-guided venous access after being taught by STs (Table 2). Based on these scores, students were able to competently perform MSK US to guide an IV-access procedure. In addition, 83.3% to 100% of students surveyed agreed that they knew how to position, angle, and use US probes to gain venous access (Figure 1). The majority (82.4%) of students agreed that they felt confident using basic US after training by STs in our workshops (Figure 2). Based on the high percentages of accuracy and confidence, we found that STs were effective resources to teach MSK and vascular POCUS. Additionally, the high percentage of students (82.5%) who felt confident after our MSK workshop corroborates with our previous study of confident students (70.8%) after a similar MSK workshop among only first-year medical students [10].

Some challenges we encountered included a limited number of US probes and STs to teach all the students who signed up for the workshops, further complicated by not being able to accommodate all students’ schedules for the one-hour workshops. Our solution was to expand to multiple sessions during the workshop day, allowing us to teach all students with our current resources while also giving students more flexibility to schedule their sessions. We also tried to align our workshop content with the preclinical system-based curriculum whenever possible to increase relevance and interest in the POCUS workshops. Another challenge as mentioned in the feedback was the use of single-sized needles during the US-guided intravenous access workshop. We were also limited in our findings since we only had data from our MSK and vascular US workshops. In the future, we hope to use different needle sizes and standardized patient body types as well as collect data across various US workshops to increase student US versatility and better our understanding of ST-run US workshop effectiveness across different US disciplines.

There are no universal guidelines on training US STs as of now. However, many medical schools implement similar hands-on training in small groups with faculty. These US training programs usually use either a structured course curriculum with set timelines and US topics or a self-paced individualized curriculum similar to our workshop-based training. Previous literature details that structured and self-paced US curricula can be equally effective at training medical students to become STs in basic US skills, and different types of structured versus self-paced US curricula may be best determined according to each school’s situation [11]. Our ST training during this study was a mix of both structured curriculum in the beginning for two months and then self-paced according to the US workshops our STs planned throughout the year. All training sessions were hands-on in three- to five-person groups with US faculty.

As the use of POCUS in medicine is expanding in scope and frequency, the incorporation of POCUS education into medical school is more pressing than ever [12,13]. With a solid basic science medical foundation and strong motivation to become skilled clinicians, medical students are strong candidates to develop and carry out POCUS educational programs while alleviating some of the human resources constraints the expansion of such programs would demand. Our medical school is currently in the process of expanding an official clinical skills US curriculum and currently has an optional US scholars program driven by the STs who ran the POCUS workshops to aid with curriculum development and implementation.

By developing and implementing POCUS workshops in conjunction with school administration and student interest groups, we generated strong interest in POCUS education in both the school administration and student body, discovered possible avenues of developing more effective POCUS education, and encouraged an official POCUS curriculum while training students in practical techniques and clinical application of POCUS for their future careers.

## Conclusions

The ST-run POCUS workshops were a unique way to adapt to a lack of US curriculum and a limited number of US faculty and US probes. Through the workshops, we showed the effectiveness of STs in MSK and vascular US. Moreover, the POCUS workshops provided a self-sustaining ST-run program without the need for extra funding or extra US faculty from the school, allowing us to build a US foundation within our medical school without having to postpone hands-on medical education due to limited resources. The program was an alternative approach that encouraged students to take leadership positions, be involved with the medical curriculum, and eventually participate as paid tutors in formal clinical skills US education.
